# Detection of *Toxoplasma gondii* oocysts in fresh vegetables and berry fruits

**DOI:** 10.1186/s13071-020-04040-2

**Published:** 2020-04-08

**Authors:** Cláudia S. Marques, Susana Sousa, António Castro, José Manuel Correia da Costa

**Affiliations:** 1 Centre for the Study in Animal Science (ICETA), University of Porto, Porto, Portugal; 2 Centre for Parasite Biology and Immunology, Department of Infectious Diseases, National Health Institute Dr Ricardo Jorge (INSARJ), Rua de Alexandre Herculano, 321, 4000-055 Porto, Portugal

**Keywords:** *Toxoplasma gondii*, Oocysts, Immunomagnetic separation, PCR, Vegetables, Berry fruits

## Abstract

**Background:**

*Toxoplasma gondii* is the third most important contributor to health burden caused by food-borne illness. Ingestion of tissue cysts from undercooked meat is an important source of horizontal transmission to humans. However, there is an increasing awareness of the consumption of fresh fruit and vegetables, as a possible source for oocyst transmission, since this stage of the parasite can persist and remain infective in soil and water for long time. Herein, we outline findings related with detection of *T. gondii* oocysts in vegetables and berry fruits, which are usually raw consumed. The procedure includes the estimation of the number of oocysts.

**Methods:**

Food samples were collected from local producers and supermarket suppliers. *Toxoplasma gondii* oocysts were concentrated after washing the samples by applying high resolution water filtration and immunomagnetic separation (method 1623.1: EPA 816-R-12-001-Jan 2012), in order to (i) remove potential *Cryptosporidium* spp. oocysts and *Giardia* spp. cysts present in the samples; and (ii) select *T. gondii* oocysts. *Toxoplasma gondii* oocyst detection and an estimation of their numbers was performed by conventional PCR and real time qPCR, using specific primers for a 183-bp sequence of the *T. gondii* repetitive DNA region. All PCR-positive DNA samples were purified and sequenced. Restriction enzyme digestion with *Eco*RV endonuclease confirmed the presence of the *T. gondii* DNA fragment. In addition, the presence of the parasite was observed by fluorescent microscopy, taking advantage of the oocysts autofluorescence under UV light.

**Results:**

Forty percent of the analysed samples (95% CI: 25.5–56.5%) presented the expected PCR and digested DNA fragments. These fragments were confirmed by sequencing. Microscopic autofluorescence supported the presence of *T. gondii*-like oocysts. The estimated mean (± SE) oocyst concentration was 23.5 ± 12.1 oocysts/g, with a range of 0.6–179.9 oocysts/g.

**Conclusions:**

Our findings provide relevant evidence of contamination of fresh vegetables and berry fruits with *T. gondii* oocysts.
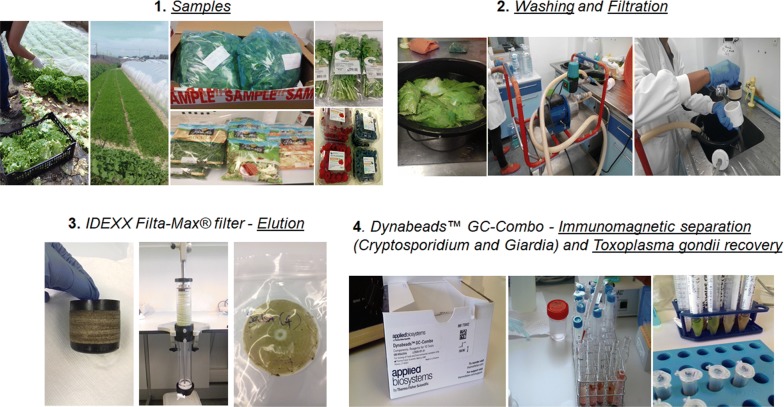

## Background

*Toxoplasma gondii* is an intracellular protozoan (Apicomplexa: Coccidia) causing human and animal toxoplasmosis [1]. Protozoan infectivity is due to three parasitic stages: an invasive tachyzoite; a bradyzoite in tissue cysts; and an environmental stage, the sporozoite, protected inside mature oocysts [1, 2]. The global prevalence of toxoplasmosis is estimated to be around 30% with 10 million clinical cases [1–4] and it is ranked as the third most important contributor to health burden caused by food-borne illness in Europe [4]. Most infections in humans are asymptomatic. However, severe complications may occur during (i) congenital *Toxoplasma* infection, such as abortion, stillbirth and hydrocephalus in new-borns [1, 4, 5]; (ii) ocular toxoplasmosis, with retinochoroidal lesions leading to chronic ocular disease [1, 2]; (iii) encephalitis in immunosuppressed patients [1, 4, 5]; and (iv) multivisceral toxoplasmosis due to atypical genotypes in South America [6–8]. A possible implication of *T. gondii* genetic diversity on the pathogenesis of toxoplasmosis has been postulated [9–11]. Consensually, current opinion is that the majority of horizontal transmissions to humans occurs after ingestion of tissue cysts in infected meat, or through water, raw fruit and vegetables contaminated with sporulated oocysts [2, 12, 13]. *In vitro* and *in vivo* experiments using mouse models have shown that infections due to oocysts are clinically more severe when compared to infections caused by tissue cysts (bradyzoites) [14]. However, the relative importance of transmission *via* tissue cysts *versus* oocysts is still unclear [2]. In addition, oocysts can remain viable for long periods in the environment and can resist chemical and physical treatment currently applied in water plants, including chlorination and ozone treatment [15–18]. This paved the way for an increasingly awareness related to drinking water and the consumption of raw fruit and vegetables as putative routes for oocyst transmission. So far, the detection of *Toxoplasma* oocysts in fruit and vegetables has been difficult, and no standardized methods are available. Moreover, findings outlined in the literature on this topic are scarce and/or controversial [19–26]. In this scenario, a laboratory approach was designed based on the experience gained with Method 1623.1/EPA for *Cryptosporidium* oocyst and *Giardia* cyst detection [27]. This approach involved: (i) the concentration of oocysts from large volumes of washing water (fruit and vegetables), according to Method 1623.1/EPA; and (ii) subsequent application of PCR for identification and quantification of *T. gondii* DNA [23, 26, 28–30]. Herein, we outline findings related with the detection of *T. gondii* oocysts in vegetables and berry fruits, as a contribution to a better comprehension of oocyst transmission to humans.

## Methods

### Food samples

**Fig. 1:**
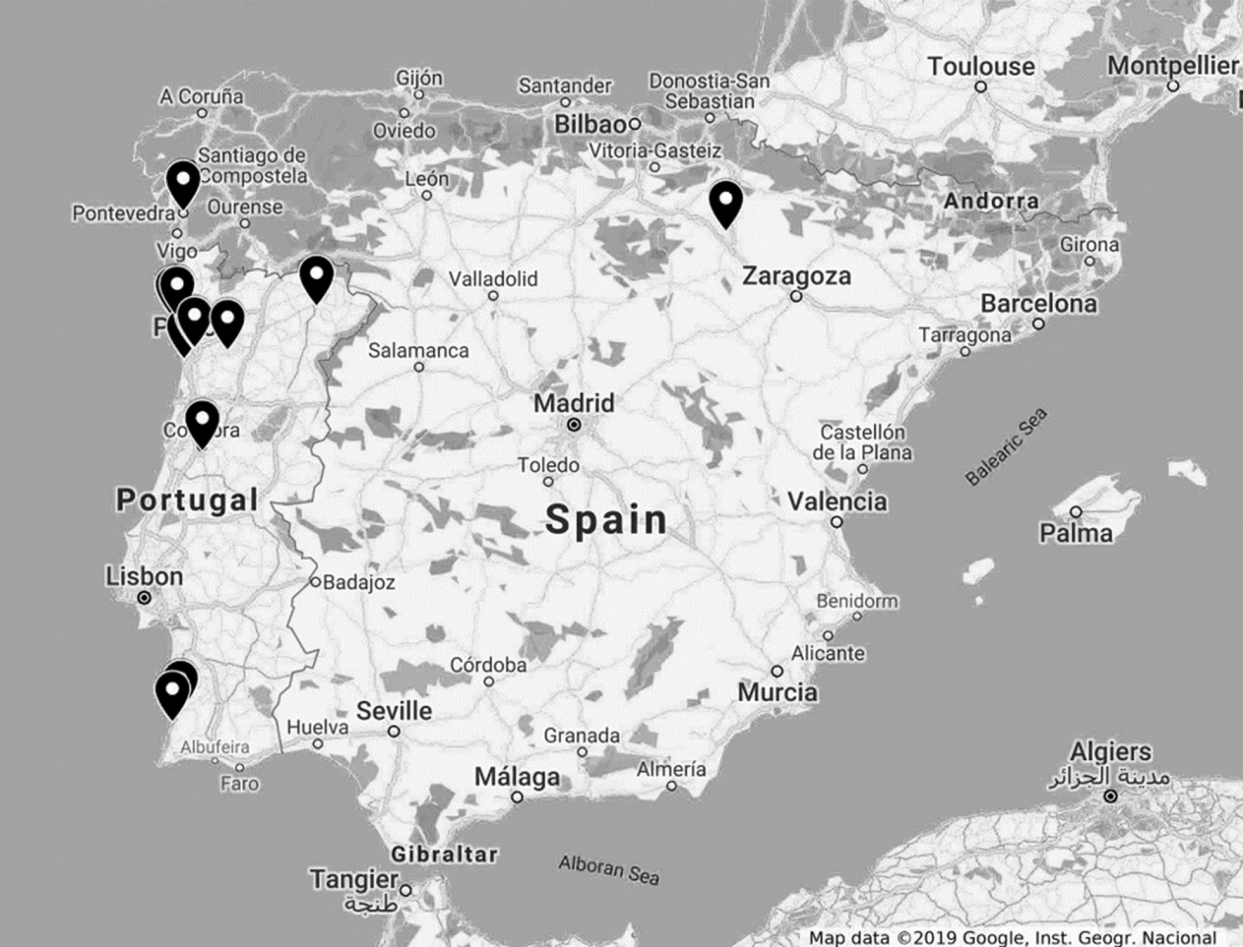
Geographical origin of the collected samples. Fruit and vegetables were collected from local producers, provided by retail sellers, or bought in small- and large-scale supermarkets in several locations of Portugal and Spain

A total of 35 bulk, packaged and ready-to-eat (RTE) vegetables and berry fruits were collected from local producers, provided by retail sellers, or bought in small and large-scale supermarkets between July 2018 and July 2019, in several locations in Portugal and Spain (Fig. 1). The choice of samples was based on expected market preferences of raw fruit and vegetables by Portuguese consumers, described by the Government agency “Instituto Nacional de Estatística” [31]. The food products included different varieties of lettuce (*Lactuca sativa*), watercress (*Nasturtium officinale*), coriander (*Coriandrum sativum*), parsley (*Petroselinum crispum*) carrots (*Daucus carota sativus*), arugula (*Eruca vesicaria sativa*), strawberries (*Fragaria ananassa*), raspberries (*Rubus idaeus*) and blueberries (*Vaccinium myrtillus*). Ready-to-eat mixed salads, with different varieties of lettuce (*Lactuca sativa*), arugula (*Eruca vesicaria sativa*), endive (*Cichorium endivia*), chicória-italiana (*Cichorium intybus*), carrots (*Daucus carota sativus*), red cabbage (*Brassica oleracea* var*. capitata* f. *rubra*) and lambʼs lettuce (*Valerianella locusta*) were also included in this study. Laboratory sample treatment was performed immediately or in less than 24 h after refrigeration.

### *Toxoplasma gondii* oocyst recovery

The concentration and recovery of *T. gondii* oocysts, as well as *Cryptosporidium* spp. oocysts and *Giardia* spp. cysts from fruit and vegetable samples were performed by Filtration/Immunomagnetic Separation (IMS)/Fluorescence Assay (FA) (Method 1623.1: *Cryptosporidium* and *Giardia* in water; US EPA 816-R-12-001-Jan 2012) [27]. Briefly, the fruit and vegetables were vigorously washed by manual swirling, for at least 10 min in large volumes of distilled water (between 10 and 80 l) in 20, 50 or 100 l tanks, accordingly to the size of each sample. Ten litres of distilled water were used to wash an average of 440 g of sample. The weight of each sample varied between 64–3600 g (Table 1); for washing water filtration, a 1 µm Filta-Max^®^ filter (IDEXX, Westbrook, ME, USA) applied to a peristaltic pump at three bar was used. Elution was performed in a Filta-Max^®^ manual wash station, and concentrated into 3 ml of phosphate-buffered solution (PBS) with 0.01% of Tween 20 (Merck KGaA, Darmstadt, Germany), after centrifugation at 1000×*g* for 10 min at room temperature. Magnetic beads conjugated with specific antibodies (Dynabeads™ GC-Combo; Thermo Fisher Scientific, Waltham, MA, USA) were added to the concentrate, and magnetized *Cryptosporidium* oocysts and *Giardia* cysts were removed from the extraneous material using a magnet (data not presented). The remaining suspension, containing putative *T. gondii* (PTG) oocysts were centrifuged (1000×g for 10 min at room temperature) and resuspended in 2–5 ml of PBS.

**Table 1:** Results of *Toxoplasma gondii* oocyst detection and quantification in the collected vegetables and fruits

Sample number	Product	Weight (g)	Origin	Collection date	Agricultural system	Product presentation	DNA^a^	Enzymatic restriction confirmation	Sequence confirmation	Mean no. of oocysts/g
1	Lettuce	3600	Aguçadoura (Póvoa do Varzim)	12-Jul-2018	Conventional	Bulk	Positive	Yes	Yes	1.3
2	Carrot	924	Aguçadoura (Póvoa do Varzim)	12-Jul-2018	Conventional	Bulk	Negative	–	–	–
3	Parsley	266	Aguçadoura (Póvoa do Varzim)	12-Jul-2018	Conventional	Bulk	Negative	–	–	–
4	Lettuce	261	Aguçadoura (Póvoa do Varzim)	10-Aug-2018	Conventional	Bulk	Negative	–	–	–
5	Lettuce	261	Mabegondo-Abegondo (Galiza)	13-Aug-2018	Organic	Bulk	Positive	Yes	Yes	3.6
6	Lettuce	600	Gondomar (Porto)	13-Aug-2018	Organic	Bulk	Positive	Yes	Yes	2.8
7	Strawberry	64	São Félix da Marinha (Vila Nova de Gaia)	05-Sep-2018	Organic	Bulk	Positive	Yes	Yes	8.6
8	Watercress	792	Boavista dos Pinheiros (Odemira)	27-Sep-2018	Conventional	Bulk	Positive	Yes	Yes	0.6
9	Lettuce	1246	Boavista dos Pinheiros (Odemira)	27-Sep-2018	Conventional	Bulk	Positive	Yes	Yes	1.1
10	Lettuce	2031	Boavista dos Pinheiros (Odemira)	27-Sep-2018	Conventional	Bulk	Negative	–	–	–
11	Coriander	749	Boavista dos Pinheiros (Odemira)	27-Sep-2018	Conventional	Bulk	Negative	–	–	–
12	Parsley	632	Boavista dos Pinheiros (Odemira)	27-Sep-2018	Conventional	Bulk	Negative	–	–	–
13	Watercress	900	Boavista dos Pinheiros (Odemira)	02-Oct-2018	Conventional	RTE	Negative	–	–	–
14	Mix salad	800	Boavista dos Pinheiros (Odemira)	02-Oct-2018	Conventional	RTE	Negative	–	–	–
15	Coriander	450	Boavista dos Pinheiros (Odemira)	02-Oct-2018	Conventional	Packaged	Positive	Yes	Yes	3.9
16	Parsley	450	Boavista dos Pinheiros (Odemira)	02-Oct-2018	Conventional	Packaged	Positive	Yes	Yes	44.5
17	Watercress	900	Boavista dos Pinheiros (Odemira)	12-Oct-2018	Conventional	RTE	Positive	Yes	Yes	3.2
18	Mixed salad	700	Boavista dos Pinheiros (Odemira)	12-Oct-2018	Conventional	RTE	Positive	Yes	Yes	6.0
19	Coriander	350	Boavista dos Pinheiros (Odemira)	12-Oct-2018	Conventional	Packaged	Positive	Yes	Yes	38.3
20	Parsley	350	Boavista dos Pinheiros (Odemira)	12-Oct-2018	Conventional	Packaged	Negative	–	–	–
21	Mixed salad	400	Boavista dos Pinheiros (Odemira)	04-Feb-2019	Conventional	RTE	Positive	Yes	Yes	179.9
22	Lettuce	494	Torre de Bera (Coimbra)	04-Mar-2019	Organic	Bulk	Negative	–	–	–
23	Lettuce	1100	Torre de Bera (Coimbra)	04-Mar-2019	Organic	Bulk	Negative	–	–	–
24	Strawberry	500	Macedo de Cavaleiros (Bragança)	27-May-2019	Conventional	Bulk	Positive	Yes	Yes	2.5
25	Strawberry	500	Macedo de Cavaleiros (Bragança)	27-May-2019	Conventional	Bulk	Positive	Yes	Yes	3.1
26	Mixed salad	800	Milagro (Navarra)	17-Jun-2019	Conventional	RTE	Negative	–	–	–
27	Mixed salad	800	Milagro (Navarra)	17-Jun-2019	Conventional	RTE	Negative	–	–	–
28	Arugula	500	Milagro (Navarra)	17-Jun-2019	Conventional	RTE	Negative	–	–	–
29	Raspberry	500	Brejão (Odemira)	17-Jun-2019	Conventional	Packaged	Negative	–	–	–
30	Blueberry	500	Laundos (Póvoa do Varzim)	17-Jun-2019	Conventional	Packaged	Negative	–	–	–
31	Lettuce	250	São Pedro da Cova (Gondomar)	18-Jun-2019	Organic	Bulk	Negative	–	–	–
32	Lettuce	270	São Pedro da Cova (Gondomar)	18-Jun-2019	Organic	Bulk	Positive	Yes	Yes	53.3
33	Mixed salad	800	Milagro (Navarra)	15-Jul-2019	Conventional	RTE	Negative	–	–	–
34	Blueberry	625	Santa Leucádia (Baião)	15-Jul-2019	Organic	Packaged	Negative	–	–	–
35	Raspberry	625	Santa Leucádia (Baião)	15-Jul-2019	Conventional	Packaged	Negative	–	–	–

^a^529-bp REP

*Abbreviations*: REP: repetitive region; RTE: ready-to-eat

### *Toxoplasma gondii* DNA detection and oocysts number estimation

#### DNA extraction

Two-hundred microliters of PTG suspension was centrifuged at 1000×*g* for 10 min at room temperature and the pellet resuspended in 200 ml lysis buffer (QIAamp^®^ DNA Mini Kit; Qiagen, Hilden, Germany). The disruption of the oocyst cell wall was performed with 4 freeze (− 20 °C)/thaw (95 °C) cycles. Prior to overnight incubation at 56 °C with 20 µl proteinase K (Qiagen), samples were treated with 1 ml of InhibitEX buffer (Qiagen) that efficiently removes PCR inhibitors commonly present in environmental samples. The commercial kit, QIAamp^®^ DNA Mini Kit (Qiagen), was used for DNA isolation according to the manufacturer’s instructions.

#### Conventional PCR and sequencing

Specific primers (FW: 5′-AGC CAC AGA AGG GAC AGA AG-3′ and REV: 5′-TCC AGG AAA AGC AGC CAA G-3′) targeting a 183-bp sequence of the 529-bp repetitive sequence of *T. gondii* [23, 26, 28–30] were designed (Primer3Plus and BLAST^®^) for DNA detection according to the MIQE (minimum information for publication of quantitative real-time PCR experiments) guidelines [32, 33]. These primers do not bind to any site of the *H. hammondi* 529-bp repetitive sequence (GenBank: EU493285.1), and no significant similarity was found between the primers’ nucleotides and *H. hammondi* repetitive sequence. PCR amplification was performed with an initial polymerase activation step (3 min at 95 °C), followed by 35 cycles of denaturation (30 s at 95 °C), annealing (30 s at 62 °C) and extension (30 s at 72 °C), followed by a final extension step of 10 min at 72 °C. The amplification reaction mixture consisted of 12.5 μl DreamTaq™ Hot Start Green PCR Master Mix 2× (Thermo Fisher Scientific), 600 nM of each primer (Eurofins Genomics, Ebersberg, Germany) and 5 μl of template DNA in a 25 μl reaction volume. Amplifications were performed in a C1000 Touch™ thermal cycler (Bio-Rad, Hercules, CA, USA). PCR products (183 bp) were observed in a Gel DocTM XR+ (Bio-Rad) and analysed using Image LabTM Software (Bio-Rad). In all PCR experiments, a positive control (genomic DNA isolated from 10^5^*T. gondii* ME49 oocysts) and a negative control (water template) were used. Additionally, all of the negative samples were retested for the presence of PCR inhibitors by adding 1 μl of *T. gondii*-positive control to the 5 μl of DNA template. Positive PCR fragments were purified (IllustraTM GFXTM PCR DNA and Gel Band Purification kit; GE HealthCare, IL, Chicago, USA) from a low melting 2% agarose gel (Lonza, Basel, Switzerland) and sequenced using Sanger sequencing services from GATC Biotech (Eurofins Genomics, Ebersberg, Germany). Sequence comparison was made with already published sequences (GenBank: AF146527.1) using the NCBI Basic Local Alignment Search Tool (BLAST).

#### Quantitative real-time PCR

A quantitative (qPCR) method based on a standard curve was used to estimate the initial number of oocysts per gram of vegetable or fruit. Briefly, a standard template was obtained after purification of the positive control (*T. gondii* ME49 oocysts) PCR product and DNA concentration was measured with a NanoDrop 1000 spectrophotometer (ThermoFisher Scientific, Waltham, MA, USA). The DNA copy number/µl was calculated and two series of a ten-fold serial dilution of the DNA (from 1 × 10^6^ to 1 copy/μl) were prepared. qPCR was carried out in a final volume of 20 µl, using 10 μl of SsoAdvanced™ Universal SYBR^®^ Green Supermix (2×) (Bio-Rad) and 350 nM of each specific primer (previously used for conventional PCR) and 2 μl of the DNA sample or standard dilution. The plates were sealed and centrifuged at 1000×*g* for 1 min, at room temperature. The cycling conditions for the qPCR CFX96 Touch real-time instrument (Bio-Rad) were as follows: initial denaturation at 98 °C for 3 min; followed by 35 cycles at 98 °C for 15 s; then 62 °C for 30 s. Melting curve analysis was also performed at the end of each PCR run (65–95 °C at 0.5 °C/5 s). qPCR of the standard dilution gradient and the DNA isolated from fruit and vegetable samples were run in duplicate and in the same plate. The absolute quantification of the initial DNA copy number/μl and estimation of the number of oocysts per gram of each environmental sample was calculated by comparing the quantification cycle threshold (Cq) values of the environmental samples with the standard curve taking into account the variation of the 529-bp repetitive sequence and the ploidy of the *T. gondii* oocysts.

#### Restriction enzyme digestion

Purified positive PCR fragments were subject to restriction enzyme digestion with the endonuclease *Eco*RV (New England Biolabs, Ipswich, MA, USA). The reaction was performed in a volume of 10 μl with 5 μl of DNA, 1 μl of the enzyme and 1 μl of 10× NEBuffer 3.1, according to the manufacturer’s instructions. Each sample was incubated at 37 °C for 1 h. Additionally, purified *T. gondii* PCR fragments and uncut experimental DNA were used as controls. The digested fragments (74 bp and 109 bp) were electrophoresed on a 2% high-resolution agarose gel (Sigma-Aldrich) and analysed using a Gel DocTM XR+ (Bio-Rad).

### Epifluorescence microscopy

Twenty-five microliters of the PTG suspension was diluted (1:2) and air-dried onto a 2-well SuperStick™ Slide (Waterborne™, Inc., New Orleans, LA, USA) chemically treated to increase adhesion of the oocysts. Fixation was performed for 2 min with absolute methanol (Panreac Química SLU, Castellar del Vallès, Spain) and the slides were allowed to dry completely. As a positive control, a slide was prepared with 10^5^*T. gondii* ME49 oocysts (kindly provided by J. T. Dubey). The background fluorescence was reduced by adding Evans Blue dye solution (BlockOut Counterstain; Waterborne™, Inc.), slides were rinsed with PBS and completely air-dried before being mounted with DPX mounting media (Merck KGaA, Darmstadt, Germany). Oocysts were visualized using a Nikon fluorescence microscope (Nikon Optiphot 2; Melville, NY, USA), under bright field and ultraviolet (UV) filter block (excitation 335 nm; emission 450 nm), based on the autofluorescent nature of the oocyst wall due to Tyr-rich proteins [18, 34]. Images were captured at 400× magnification using a PowerShot A630 digital camera (Canon, Amstelveen, Holland).

### Statistical analysis

Graphpad Prism version 7.02 (San Diego, CA, USA) was used for statistical analysis. The 95% confidence intervals (95% CI) for the population proportions were calculated using the modified Wald method [35]. Fisher’s exact test was used to find out differences for the different categorical variables. The non-parametric Mann-Whitney U-test was used to compare the number of oocysts per gram in each food group. A two-tailed *P-*value of less than 0.05 was considered significant.

## Results

### Sampling

A total of 25 kg of fresh products distributed in 35 samples were analysed and results are shown in Table 1. These comprised 18 bulk samples, corresponding to 14.5 kg and 17 samples of packaged and RTE corresponding to 10.5 kg; 28 samples (21.7 kg) of fresh vegetables (lettuce, watercress, parsley, coriander, arugula, carrots and mixed salads) and 7 (3.3 kg) of berry fruits (strawberry, raspberry and blueberry); 27 samples (21.3 kg) were provided from conventional agriculture systems and 8 samples (3.7 kg) from organic agriculture.

### *Toxoplama gondii* DNA detection

**Fig. 2:**
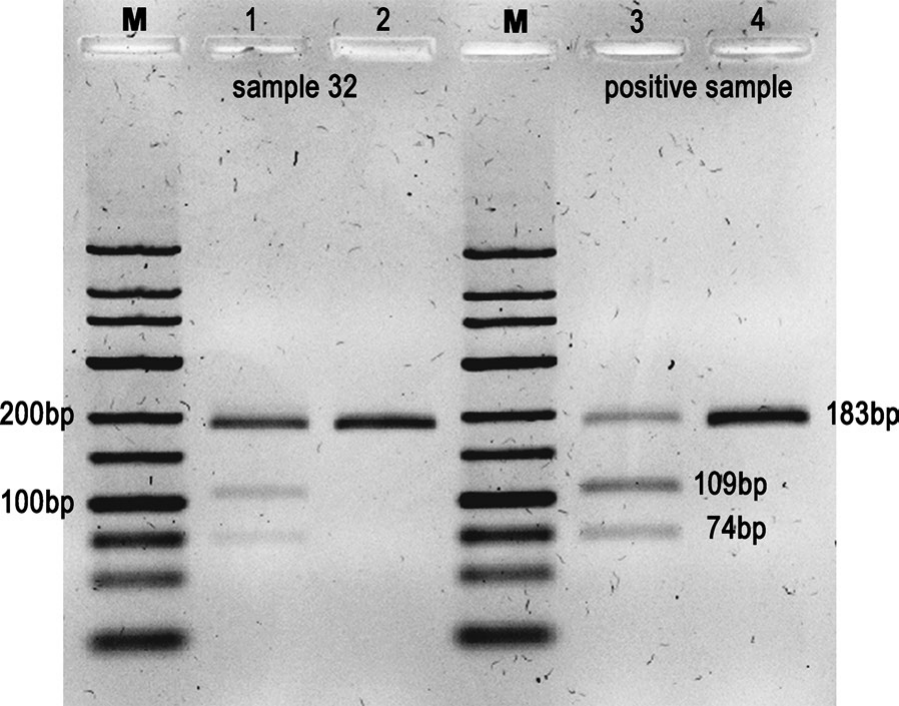
Representative image of a 2% high resolution agarose gel with *T. gondii* uncut, positive, 183 bp PCR fragments (Lane 2 and Lane 4) and subject to restriction enzyme digestion with the endonuclease *Eco*RV (Lane 1 and Lane 3). A ready-to-use DNA marker (Lanes M) suitable for sizing linear double-stranded DNA fragments from 25–700 bp was used to confirm the expected digested fragments of 74 bp and 109 bp

*Toxoplasma gondii* detection was performed by conventional PCR and a 183-bp gel band corresponding to the specific DNA fragment of the *T. gondii* repetitive region (GenBank: AF146527.1) was observed in 14 out of the 35 (40.0%) analysed samples (95% CI: 6.5–25.5%). Statistical analysis of contingency tables showed no significant differences (*P* > 0.05) for the different categorical variables, such us the type of products, the season when they were collected, the type of agriculture system applied and Bulk or RTE products (Table 2). Sequence analysis confirmed that the obtained amplicon of all positive samples had more than 95% nucleotide similarity with the 529-bp *T. gondii* repetitive region (GenBank: AF146527.1) (Table 1). Moreover, two bands of expected sizes (109 bp and 74 bp) were observed after *Eco*RV DNA enzymatic digestion (Fig. 2), confirming *T. gondii* DNA sequence.

**Table 2:** Prevalence rate of *Toxoplasma gondii* in different groups of fruit and vegetables

Samples	*Toxoplasma gondii* DNA detection		*P-*value
	Negative (*n*, %)	Positive (*n*, %)	
Total	21 (60.0)	14 (40.0)	
Product presentation			
Bulk	9 (50.0)	9 (50.0)	*P* = 0.499
RTE/packaged	11 (64.7)	6 (35.3)	
Type of product			
Vegetables	16 (57.1)	12 (42.9)	*P* = 1.000
Berry fruits	4 (57.1)	3 (42.9)	
Agricultural system			
Conventional	16 (59.3)	11 (40.7)	*P* = 0.700
Organic	4 (50.0)	4 (50.0)	
Season			
Spring/summer	12 (63.2)	7 (36.8)	*P* = 0.506
Autumn/winter	8 (50.0)	8 (50.0)	

*Notes*: Contingency tables were constructed in order to compare categorical variables. The Fisher’s exact test was used and *P* > 0.05 indicates non-significant associations

*Abbreviations*: *n*, number of samples; %, percentage

### Estimation of the number of *T. gondii* oocysts

**Fig. 3:**
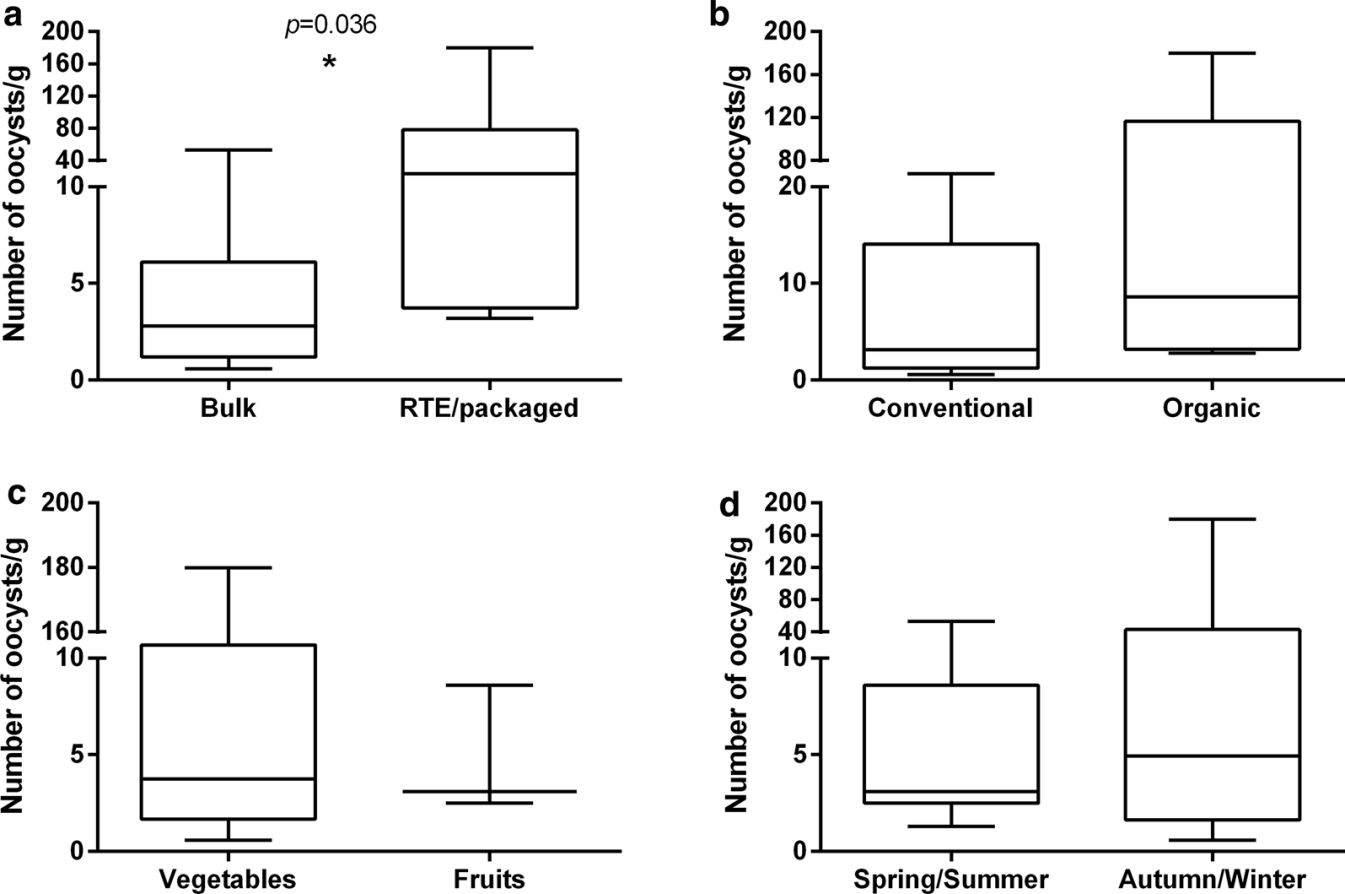
Box and whiskers diagrams comparing the estimated number of oocysts per gram, between bulk and RTE/packaged products (**a**), conventional and organic agriculture system (**b**), fruit and vegetables (**c**) and food items collected in spring/summer and autumn/winter (**d**), quantified by qPCR. The non-parametric Mann-Whitney U-test was used to compare the estimated number of oocysts per gram between the groups. *P = 0.036

Real-time qPCR was used to estimate the initial number of oocysts present in each gram of vegetable or fruit. Quantification was performed by using as a standard, a *T. gondii* DNA gradient of a known initial number of DNA copies/µl. Amplification efficiencies were calculated and were always between 90–110%. In addition, melt curve analysis allowed confirmation of the specific *T. gondii* amplified sequence. The diagnostic melting temperature (Tm) peak was 86.5 ± 1 °C. The concentration of *Toxoplasma* from the fruit and vegetables varied between 0.6–179.9 oocysts/g (Table 1), with a mean number (± SE) of 23.5 ± 12.1 oocysts/g. A significantly higher number of oocysts/g was found from RTE/packaged samples (45.9 ± 27.8 oocysts/g) when compared with the mean number of oocysts/g present in bulk products (8.5 ± 5.6 oocyst/g) (Mann-Whitney U-test: *P* = 0.036) (Fig. 3a). No statistically significant differences (*P* > 0.05) were found when comparing the number of oocysts/g in products originally from conventional or organic agriculture, between fruit and vegetables or the season when the products were collected (Fig. 3b–d).

### *Toxoplasma gondii* oocysts autofluorescence

Direct visualization of *T. gondii* oocyst autofluorescence was performed using an epifluorescent microscope. In slides from all PCR-positive samples we observed *T. gondii*-like oocysts, based on morphology and size (between 10–15 µm in diameter), and the pale blue glow when illuminated with a UV light source. The majority of the samples presented unsporulated oocysts. However, sporulated oocysts were observed in one of the strawberry samples.

## Discussion

Water and food matrices may be accidentally contaminated by environmental oocysts [12, 13]. Thus far, there are no standard methods or consensual tools described for oocyst identification and quantification from these matrices. The lack of standardised methods probably sustains the exclusion of *T. gondii* from regular surveillance systems [12, 13, 26]. In order to estimate the prevalence of *T. gondii* oocysts in fresh fruit and vegetables, we have designed an approach described herein. For molecular detection of the parasite, we have selected a consensual specific DNA fragment of the *T. gondii* repetitive region [28]. The 529-bp DNA fragment was amplified in 40% of all samples, with an estimated mean of 23.5 oocysts per gram of product. Our findings indicate a higher prevalence compared to other studies [19–26]. However, critical analysis of the data in Table 3, evidences major differences related to: (i) sampling design; (ii) DNA target strategy; and of relevance to this particular study (iii) the choice of oocyst recovery method. In our opinion, the choice of the Filta-Max^®^ System (IDEXX), for high-resolution filtration of large volumes of washing water, increases oocyst recovery. Consequently, it increases the sensitivity of the detection method. Complementary to this, the execution of Method 1623.1 [27] seems to be an advisable strategy for removing *Cryptosporidium* spp. oocysts and *Giardia* spp. cysts and separating them from potential *T. gondii* oocysts present in the sample washing water. Indeed, high-grade filtration followed by immunomagnetic separation of water samples has been described as a more accurate method for parasite concentration when compared with membrane filtration and centrifugation [36] or sucrose gradient separation [37].

**Table 3:** Current available data on the detection of *Toxoplasma gondii* oocysts in fresh fruit and vegetables, under non-experimental conditions

References	Product	Origin	Sample size	Oocyst recovery method	Detection method	PCR target	*T. gondii* prevalence (%)	No. of oocysts	Sequence confirmation	Genotyping analysis
Al-Megrin (2010) [19]	Spinach, radish, leek, parsley, basil, green onion, dill, lettuce, cabbage, watercress, coriander, mint	Saudi Arabia	*n* = 470 (250 g)	Washing, centrifugation	Microscopy	na	6.6	nd	No	No
Lass et al. (2012) [20]	Lettuce, strawberries, carrots, radishes	Poland	*n* = 216 One lettuce; 1000 g strawberries; 500 g carrots; 20 radishes	Washing, centrifugation, flocculation (CaCO3)	Real-time qPCR	B1 gene	9.7	1–20/sample	No	Yes (type I and II)
Haq et al. (2014) [21]	Cabbage, carrot, chili, coriander, cucumber, lettuce, mint, radish, tomato	Pakistan	*n* = 500 (200–250 g)	Washing, centrifugation, sedimentation or flotation	Microscopy	na	1.9	nd	No	No
Lalonde & Gajadhar (2016) [22]	Pre-packaged or bulk spinach, lettuce, chard, collards, dandelion greens, rapini, mixed salads	Canada	*n* = 470 (250 g)	Washing, centrifugation, flotation (Sheather’s sucrose solution)	Real-time PCR	*18S* rDNA	0.26	nd	Yes	No
Marchioro et al. (2016) [23]	Organic and nonorganic: crisp lettuce, regular lettuce, chicory, rocket, parsley	Brazil	*n* = 1171 (35 g)	Washing, filtration (0.3 mm cellulose ester membrane), centrifugation	Conventional PCR	B1 gene/ 529 bp REP	2.9/0.8	nd	No	No
Caradonna et al. (2016) [24]	Ready-to-eat packaged salads	Italy	*n* = 238 (50 g)	Washing, centrifugation	Microscopy, qPCR, sequencing	B1 gene	0.8	62–554/g	Yes	Yes (type I)
Ferreira et al. (2018) [25]	Crisp lettuce, arugula, chicory, chives, purple lettuce, spinach, chard	Brazil	*n* = 470 (250 g)	Washing, filtration (two layers of gauze), centrifugation	Conventional PCR	529 bp REP	12.9	nd	No	No
Slany et al. (2019) [26]	Carrot, cucumber, lettuce	Czech Republic	*n* = 648 (100 g)	Washing, centrifugation	Triplex qPCR	B1 gene and 529 bp REP	9.6	1–900/g	No	Type II
Present study	RTE/packaged and bulk (organic and nonorganic: lettuce, watercress, coriander, parsley, carrots, arugula, strawberries, raspberries, blueberries, mixed salads)	Portugal, Spain	*n* = 35 (64–3600 g)	IDEXX Filta-Max® filtration EPA method1623.1	Microscopy, conventional PCR, qPCR, restriction enzyme, sequencing	529 bp REP	40.0	1–180/g	Yes	No

*Abbreviations*: na, not applicable; nd, not determined; REP, repetitive region

In our study, *T. gondii* DNA fragments were confirmed by sequencing and by DNA digestion with a restriction enzyme. Enzymatic digestion with *Eco*RV endonuclease suggests specificity and excludes cross-amplification of non-target organisms, such as *Hammondia hammondi*; this closely related apicomplexan presents a homologous region with around 84% sequence identity with the 529-bp repeat region of *T. gondii* [38]. The selected endonuclease *Eco*RV does not bind to any recognition site of the *H. hammondi* repetitive sequence, consequently, DNA cleavage does not occur.

Slides from PCR-positive samples were examined under epifluorescent microscopy and autofluorescent structures, compatible with *T. gondii* unsporulated oocysts in morphology and size (between 10–15 µm in diameter) were observed. A sporulated oocyst was observed in one sample. Data concerning the direct microscopic examination of oocysts in fresh vegetables are scarce [19, 21]. Indeed, no clear references to the observation of *T. gondii* sporulated oocysts has yet been described in fruit or vegetables. On the other hand, *T. gondii* oocysts are morphologically indistinguishable from the oocysts of related coccidian parasites, not infectious to humans, such as *Hammondia* spp. and *Neospora caninum* [13]; debris present in the samples may affect the ability to visualize small fluorescent structures such as autofluorescent oocysts, and so far there are no commercial antibodies to implement a specific immunomagnetic separation protocol. Therefore, and in the absence of standard methods for oocysts observation, *T. gondii* confirmation always requires molecular identification. Our findings suggest no significant seasonal variation or differences between the type of agriculture system applied. Additionally, no significant differences between the prevalence of *T. gondii* in the leafy vegetables or fruits were observed. Although a statistically significant difference in the proportion, has not been observed between bulk/RTE and positive *T. gondii* DNA amplification, curiously, a remarkably higher concentration of *Toxoplasma* oocysts was found in RTE and packaged fruit and vegetables, when compared to bulk products. So far, we have no clear explanation for this result. However, it is reasonable to speculate about the management of the washing systems used in food industry. Trevisan et al. [39] referred that managing water sustainably in agriculture means increased utilization of wastewater for irrigation, reusing freshwater to wash the products and therefore increasing the chances for RTE/packaged fruit and vegetables to become contaminated [39]. Nevertheless, testing water samples from washing/packing facilities and the water used for irrigation of bulk products are warranted in order to decipher the degree of *Toxoplasma* oocyst contamination on fruit and vegetables. In addition, genotyping should be carried out in all positive-PCR samples, for risk assessment evaluation.

## Conclusions

Our findings sustain that consumption of raw fruit and vegetables may be a source of *T. gondii* infection in humans. They also emphasise the need of a procedure accepted with a consensus as the “gold standard” method for the recovery, detection and quantification of *T. gondii* oocysts and validated for use with fresh fruit and vegetables. Major improvements are still required for routine application at the industrial level or for food testing in laboratories for detection of *T. gondii* oocysts.

## Data Availability

Data supporting the conclusions of this article are included within the article. The datasets used and analysed in the present study are available from the corresponding author upon reasonable request.

## Abbreviations

BLAST: basic local alignment search tool
CI: confidence interval
FA: fluorescence assay
IMS: immunomagnetic separation
MIQE: minimum information for publication of quantitative real-time PCR experiments
PBS: phosphate-buffered solution
PTG: putative *T. gondii*
qPCR: quantitative PCR
RE: restriction enzyme
RTE: ready-to-eat
SE: standard error
Tm: melting temperature
